# Towards a Unified Terminology for Implant-Influenced Fractures: Implications for Musculoskeletal and Muscle–Implant Interaction Research

**DOI:** 10.3390/muscles5010007

**Published:** 2026-01-15

**Authors:** Giacomo Papotto, Ignazio Prestianni, Enrica Rosalia Cuffaro, Alessio Ferrara, Marco Ganci, Calogero Cicio, Alessandro Pietropaolo, Marco Montemagno, Saverio Comitini, Antonio Kory, Rocco Ortuso

**Affiliations:** 1 Department of Surgery, Giovanni Paolo II Hospital, 92019 Sciacca, Italy; ignazioprestianni93@gmail.com (I.P.); enricacuffaro@outlook.it (E.R.C.); alessio.ferrara1993@gmail.com (A.F.); ciciocalogero@gmail.com (C.C.); 2 Department of Orthopedic Surgery, Trauma Center, Cannizzaro Hospital, 95100 Catania, Italy; mrcganci@gmail.com (M.G.); saveriocomitini@gmail.com (S.C.); antonio.kory@gmail.com (A.K.); drrocco@libero.it (R.O.); 3 Department of General Surgery and Medical Surgical Specialties, Section of Orthopaedics, A.O.U. Policlinico Rodolico-San Marco, University of Catania, 95123 Catania, Italy; alessandro.pietropaolo@gmail.com (A.P.); docmontemagno@gmail.com (M.M.)

**Keywords:** artificial fracture, implant-related fracture, periprosthetic fracture, orthopedic terminology, medical education, musculoskeletal system, muscle–bone interaction, implant biomechanics

## Abstract

**Background:** The global increase in orthopedic implant use—both for trauma fixation and arthroplasty—has profoundly transformed musculoskeletal surgery. As a consequence, fractures occurring in the presence of implants have become more frequent and clinically relevant. Yet, these injuries are currently described using highly heterogeneous terminology, including periprosthetic (fracture occurring in the presence of a prosthetic joint replacement) peri-implant (fracture occurring around an osteosynthesis or fixation device), implant-related, and hardware-related fractures (umbrella terms encompassing both prosthetic and fixation devices, used descriptively rather than classificatorily). This coexistence of multiple, context-specific terminologies hinders clinical communication, complicates registry documentation, and limits research comparability across orthopedic subspecialties. Because fractures occurring in the presence of orthopedic implants significantly alter load transfer, muscle force distribution, and musculoskeletal biomechanics, a clear and unified terminology is also relevant for muscle-focused research addressing implant–tissue interaction and functional recovery. **Objective:** This systematic review aimed to critically analyze the terminology used to describe fractures influenced by orthopedic implants, quantify the heterogeneity of current usage across anatomical regions and publication periods, and explore the rationale for adopting a unified umbrella term—“artificial fracture.” **Methods:** A systematic search was performed in PubMed, Scopus, and Web of Science from January 2000 to December 2024, following PRISMA guidelines. Eligible studies included clinical investigations, reviews, registry analyses, and consensus statements explicitly employing or discussing terminology related to implant-associated fractures. Data were extracted on publication characteristics, anatomical site, terminology employed, and classification systems used. Quantitative bibliometric and qualitative thematic analyses were conducted to assess frequency patterns and conceptual trends. **Results:** Of 1142 records identified, 184 studies met the inclusion criteria. The most frequent descriptor in the literature was periprosthetic fracture (68%), reflecting its predominance in arthroplasty-focused studies, whereas broader and more practical terms such as implant-related and peri-implant fracture were more commonly used in musculoskeletal and fixation-related research. Terminological preferences varied according to anatomical site and implant type, and no universally accepted, cross-anatomical terminology was identified despite multiple consensus efforts. **Discussion and Conclusions:** The findings highlight persistent heterogeneity in terminology describing fractures influenced by orthopedic implants. A transversal, descriptive framework may facilitate communication across subspecialties and support registry-level harmonization. Beyond orthopedic traumatology, this approach may also benefit muscle and musculoskeletal research by enabling more consistent interpretation of data related to muscle–bone–implant interactions, rehabilitation strategies, and biomechanical adaptation.

## 1. Introduction

The evolution of orthopedic surgery over the past decades has been characterized by an exponential rise in the use of implants for both reconstructive and trauma-related purposes. Total joint arthroplasties, internal fixation devices, and spinal instrumentation have dramatically expanded, with millions of procedures performed annually worldwide. This progress, while improving functional outcomes and quality of life, has also led to an increasing number of fractures occurring in the context of pre-existing implants.

Despite the growing frequency of such events, the terminology used to describe them remains heterogeneous and context-dependent across orthopedic subspecialties. The literature alternately refers to periprosthetic [1], peri-implant [2], hardware-related [3], and implant-associated [4] each reflecting distinct historical, anatomical, or disciplinary preferences. The absence of standardized terminology complicates communication among clinicians, coders, and researchers, and undermines efforts to compare outcomes across studies.

The need for a unified language has long been recognized. Since the early 2000s, international working groups such as the AO Foundation/Orthopaedic Trauma Association (AO/OTA), the International Society of Fracture Repair (ISFR), and the International Consensus Symposium (ICS) have attempted to reconcile disparate terminologies. However, these initiatives have largely focused on specific anatomical regions—most notably periprosthetic fractures around the hip and knee—leaving other areas underrepresented.

In addition to academic inconsistency, this terminological fragmentation carries significant clinical, educational, and economic implications. Variability in descriptors can influence coding accuracy, reimbursement, registry classification, and even medico-legal interpretation of postoperative complications. Furthermore, in an era increasingly dominated by electronic health records (EHRs) and machine-learning-based clinical databases, semantic heterogeneity limits interoperability and automated data retrieval.

This review seeks to systematically analyze existing terminological practices in the field, quantify the degree of inconsistency, and propose a unifying concept—the artificial fracture—as a potential cross-anatomical, clinically intuitive descriptor. We aim not to replace established classification systems but to provide a meta-category that can serve as a semantic bridge among them, fostering clarity and facilitating digital standardization.

## 2. Materials and Methods

### 2.1. The Search Strategy

We performed a systematic search in PubMed, Scopus, and Web of Science from January 2000 to December 2024. Search strings included:

- “periprosthetic fracture”
- “peri-implant fracture”
- “implant-related fracture”
- “hardware-related fracture”
- combined with anatomical terms (hip, knee, shoulder, elbow, spine, femur, tibia, humerus, forearm).

### 2.2. Inclusion/Exclusion Criteria

- Inclusion: clinical studies, reviews, registry analyses, consensus papers using or explicitly discussing fracture terminology related to implants.
- Exclusion: basic science, animal studies, biomechanical models without clinical context, case reports < 5 patients.

### 2.3. Study Selection

- Records identified: 1142

After removal of duplicates, 936 records were screened by title and abstract. Of these, 634 articles were excluded because they were clearly unrelated to fracture terminology in the context of orthopedic implants, including basic science studies, biomechanical analyses without clinical correlation, animal models, and non-orthopedic implant literature. The remaining 302 articles underwent full-text assessment for eligibility. During this phase, 118 studies were excluded for the following predefined reasons:

- absence of an explicit or consistent use of terminology related to fractures occurring in the presence of orthopedic implants;
- mixed or heterogeneous patient cohorts in which implant-related fractures could not be separately identified or analyzed;
- purely descriptive clinical reports in which fracture terminology was mentioned incidentally without conceptual or classificatory intent;
- duplicate data sets, secondary analyses, or conference abstracts lacking sufficient methodological detail.

**Figure 1:**
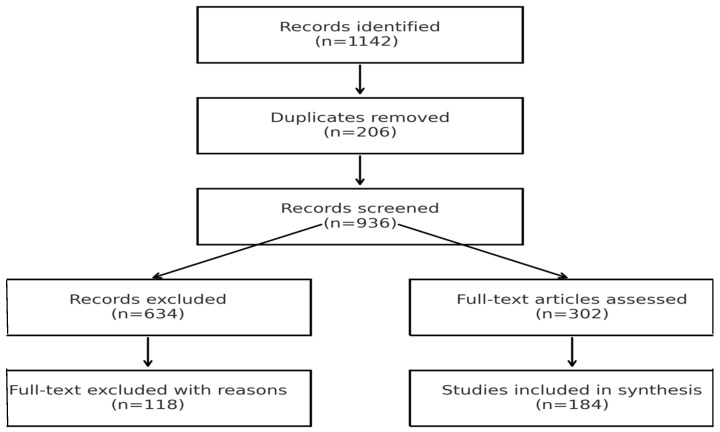
PRISMA flow diagram illustrating the systematic search and study selection process. This diagram summarizes the sequential filtering process used in accordance with the **PRISMA 2020 guidelines**, demonstrating the transparency and reproducibility of the literature search and selection workflow. The complete PRISMA checklist is provided in the Supplementary Material Table S1.

Ultimately, 184 studies met all inclusion criteria and were included in the qualitative and quantitative synthesis. (PRISMA flowchart shown in Figure 1).

### 2.4. Data Extraction and Analysis

Two reviewers independently extracted year, journal, anatomical site, terminology used, and classification system presence. Frequencies of terminology were calculated overall and by site. Trends were plotted bibliometrically. A qualitative synthesis of consensus statements was also performed.

## 3. Results

### 3.1. Overview of Study Selection

The systematic literature search identified a total of 1142 records across PubMed, Scopus, and Web of Science. After removal of duplicate entries, 936 unique records remained and were screened by title and abstract according to the predefined inclusion and exclusion criteria. Following this initial screening, 634 records were excluded.

A total of 302 articles underwent full-text assessment for eligibility. Of these, 118 studies were excluded for reasons detailed in Section 2.3, including the absence of an explicit focus on terminology related to fractures occurring in the presence of orthopedic implants or insufficient methodological detail.

Ultimately, 184 studies met all inclusion criteria and were included in the final qualitative and quantitative synthesis. The study selection process is summarized in the PRISMA flow diagram (Figure 1).

### 3.2. Temporal Trends

When analyzed across five-year intervals, the data revealed a progressive increase in publications referring to fractures associated with implants, with distinct shifts in terminology over time.

Between 2000 and 2004, the term periprosthetic fracture accounted for over 80% of publications, largely reflecting early literature focused on hip arthroplasty.

From 2005 to 2014, alternative descriptors such as implant-related fracture and peri-implant fracture began to emerge, particularly in upper-limb and spinal studies.

During 2015–2024, a growing diversification in terminology was observed, with “hardware-related” and “implant-associated” fractures increasingly used in trauma fixation contexts.

This evolution suggests a gradual recognition that fracture behavior around non-prosthetic devices warrants distinct conceptual frameworks, even though a unified terminology has not yet been consolidated.

### 3.3. Terminology Frequency

**Figure 2:**
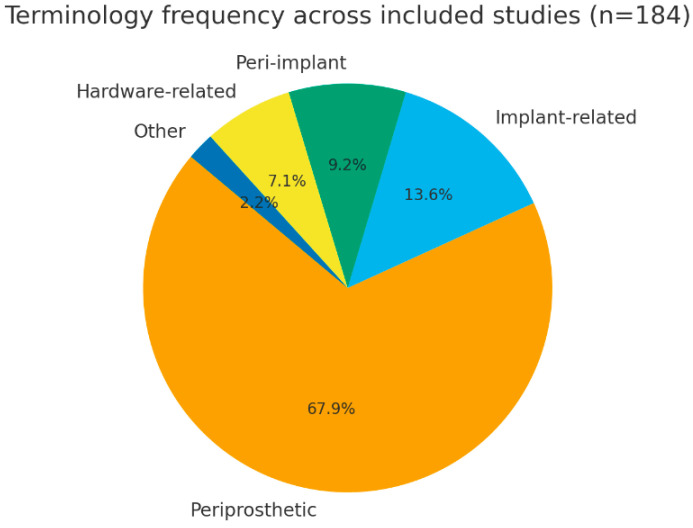
Terminology frequency.

Among the 184 included studies, the term periprosthetic fracture was the most frequently used descriptor, appearing in 125 publications (68%). This terminology was consistently applied in studies addressing fractures occurring in the presence of prosthetic joint implants, particularly in the context of hip and knee arthroplasty. Other terms were used less frequently and primarily reflected the type of implant involved rather than conceptual disagreement. Specifically, implant-related fracture was reported in 25 studies (14%), peri-implant fracture in 17 studies (9%), and hardware-related fracture in 13 studies (7%). These descriptors were predominantly employed in studies focusing on fractures associated with osteosynthesis or fixation devices, such as plates, screws, intramedullary nails, or other non-prosthetic implants. Less commonly used expressions, including implant-associated or fixation-related fracture, were identified in a limited number of publications (4 studies, 2%) and were generally used in a descriptive manner without reference to a formal classification system. Overall, the terminology adopted across the included studies was largely aligned with implant type and anatomical context, reflecting established conventions within different orthopedic subspecialties rather than inconsistent or conflicting nomenclature (Figure 2).

### 3.4. Anatomical Distribution

When stratified by anatomical region, the majority of included studies focused on fractures occurring in the presence of prosthetic joint implants of the lower limb. Specifically, fractures involving the hip accounted for 52% of the included studies, followed by the knee (19%). Upper-limb prosthetic applications, most commonly involving the shoulder, represented 11% of the publications.

Studies addressing fractures associated with spinal implants accounted for 8% of the included literature. The remaining 10% of studies focused on fractures occurring around osteosynthesis or fixation devices used for trauma management, including plates, screws, and intramedullary nails, primarily in long bones.

This anatomical distribution reflects the relative prevalence of different implant types and surgical procedures reported in the orthopedic literature rather than differences in terminological adoption or conceptual interpretation. Geographic analysis showed that most studies originated from Europe (46%) and North America (38%), with smaller contributions from Asia (14%) and Oceania (2%), consistent with established publication patterns in orthopedic research.

### 3.5. Comparative Analysis by Anatomical Site

When terminology was analyzed in relation to anatomical site, distinct usage patterns were observed across orthopedic subspecialties. In studies focusing on hip and knee arthroplasty, the term periprosthetic fracture was predominantly used, reflecting its widespread adoption in the literature addressing fractures occurring in the presence of prosthetic joint implants.

**Figure 3:**
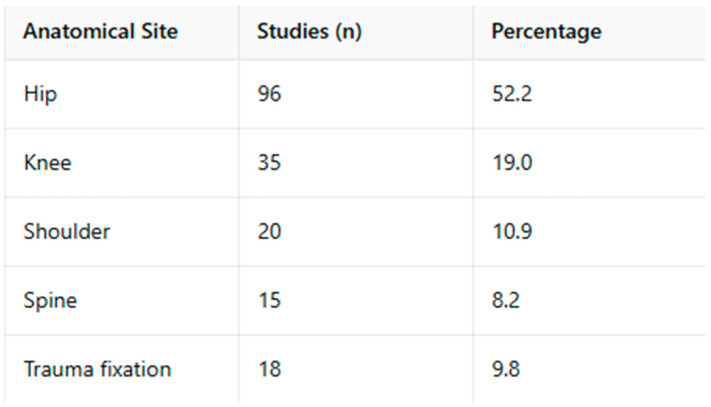
Terminology by anatomical Site.

In publications addressing the shoulder, upper limb, and spine, a broader range of terms was employed, including periprosthetic, peri-implant, and implant-related fracture. The choice of terminology varied among studies and was not consistently linked to a specific implant category, as both prosthetic and non-prosthetic devices were represented within these anatomical regions. Within the trauma and fixation literature, particularly in studies involving long bones stabilized with plates, screws, or intramedullary nails, descriptors such as peri-implant fracture and hardware-related fracture were more frequently reported. Overall, the observed variation in terminology across anatomical sites reflects differences in clinical focus and publication practices among orthopedic subspecialties, without implying anatomical inappropriateness or incorrect usage of specific terms (Figure 3).

### 3.6. Association with Classification Systems

Of the included studies, 61% referenced or applied a specific classification system, most frequently the Vancouver classification (hip, 38%), followed by the Unified Classification System (UCS) (knee and hip, 17%), and several region-specific systems such as Baba for total knee arthroplasty or Lucenti for non-prosthetic peri-implant fractures.

Interestingly, studies using standardized classifications tended to adhere more strictly to the term “periprosthetic fracture,” whereas those lacking formal classification frameworks exhibited greater lexical variability. This correlation reinforces the idea that structured classification promotes terminological consistency.

### 3.7. Consensus Group Statements

Several international initiatives have addressed fracture classification and terminology in the context of orthopedic implants, primarily with a focus on fractures occurring around prosthetic joint replacements. The AO/OTA Unified Classification System (UCS) represents one of the most widely adopted frameworks for the classification of fractures occurring in the presence of prosthetic joint implants. By design, the UCS applies to fractures associated with joint arthroplasty and does not encompass fractures occurring around osteosynthesis or fixation devices. Other consensus efforts and expert group statements have explored broader descriptive terminology for fractures occurring in the presence of orthopedic implants, particularly in the trauma and fixation literature. However, these initiatives have generally remained limited to specific anatomical regions or implant types and have not resulted in the formal adoption of a single descriptor applicable across both prosthetic and non-prosthetic implant contexts. Overall, consensus statements to date reflect a shared recognition of the diversity of implant-associated fracture scenarios, while maintaining clear conceptual boundaries between fractures related to prosthetic joint implants and those associated with fixation devices.

### 3.8. Summary of Key Findings

Terminological heterogeneity persists despite decades of discussion and the development of classification systems.

“Periprosthetic fracture” dominates in hip and knee literature, but its scope is anatomically constrained.

Broader descriptors such as “peri-implant” or “implant-related” appear in the shoulder, spine, and trauma fixation contexts, highlighting the absence of a unified lexicon.

Consensus groups acknowledge the problem but have yet to provide a universal solution, leaving an opportunity for new transversal terminology—such as the proposed concept of the “artificial fracture”—to bridge the gap.

## 4. Discussion

### 4.1. Terminology in Implant-Influenced Fractures: Current Landscape

The present review provides an overview of how fractures occurring in the presence of orthopedic implants are described across the contemporary literature. The findings indicate that terminology usage is largely consistent within specific clinical and anatomical contexts. In particular, the term periprosthetic fracture is predominantly used for fractures occurring around prosthetic joint implants [5,6], whereas descriptors such as peri-implant or hardware-related fracture are more commonly employed in the context of osteosynthesis and fixation devices [7]. Although the term periprosthetic fracture is commonly used in arthroplasty literature, contemporary musculoskeletal research frequently adopts broader terminology when addressing implant-influenced injuries across different anatomical sites.

This pattern does not reflect incorrect or conflicting terminology. Rather, it highlights the parallel evolution of language within different orthopedic subspecialties, each addressing distinct implant types, anatomical regions, and clinical priorities. Established classification systems, such as the Vancouver classification [8,9] and the AO/OTA [10] Unified Classification System (UCS), have contributed to terminological consistency within their intended scope, namely fractures associated with prosthetic joint replacements.

### 4.2. Limits of Site- and Implant-Specific Terminology

While existing terminology is internally coherent, it remains inherently site- and implant-specific. Classification systems and descriptors are designed to address local fracture patterns, biomechanics, and treatment strategies, and therefore cannot be expected to function as universal linguistic frameworks [11,12,13]. As a consequence, fractures occurring in the presence of different implant types are described using distinct terms that are not directly interchangeable, even when the underlying clinical challenge—namely, fracture behavior influenced by an implant—is conceptually similar [14,15].

This fragmentation does not represent a deficiency of current classifications, but rather reflects their intentional specificity. However, it poses challenges when implant-influenced fractures are analyzed at a higher level, such as in multicenter registries, epidemiological studies, systematic reviews, or digital health applications, where data aggregation across anatomical sites and implant types is required.

### 4.3. Rationale for a Transversal Descriptive Concept

In this context, the proposal of a transversal descriptor is not intended to replace or modify established classification systems, nor to redefine existing clinical entities. Instead, it aims to provide a meta-level conceptual category capable of encompassing all fractures whose occurrence, morphology, or management is influenced by the presence of an orthopedic implant.

The term artificial fracture is proposed as such a descriptor. The adjective “artificial” refers to the human modification of the skeletal environment through implantation of prosthetic or fixation devices, acknowledging that the biomechanical and biological behavior of fractures in this setting differs from that of native bone fractures. Importantly, this concept includes the interaction between the implant and host factors, such as bone quality, osteoporosis, or metabolic conditions, which may contribute to fracture development or complexity after implantation.

### 4.4. Definition and Scope

Within this framework, an artificial fracture can be defined as:

A fracture whose occurrence, morphology, or management is substantially influenced by the presence of an orthopedic implant and/or its interaction with host bone conditions, regardless of implant type or anatomical location.

This definition is intentionally broad and descriptive. It does not introduce a new clinical diagnosis, nor does it imply uniform treatment strategies. Instead, it serves as an overarching semantic category under which established entities—such as periprosthetic fractures and peri-implant fractures—can be conceptually grouped when required.

### 4.5. Potential Applications and Implications

The primary utility of the artificial fracture concept lies in non-clinical but increasingly relevant domains, including:

Registry and coding systems, where a transversal descriptor may facilitate consistent categorization across implant types;

Epidemiological research and meta-analyses, enabling comparison of implant-influenced fracture patterns across anatomical regions;

Digital health and artificial intelligence applications, where standardized semantic frameworks are essential for data extraction and interoperability;

Medical education, by providing trainees with a higher-order conceptual understanding of fractures occurring in altered skeletal environments.

By operating at a meta-descriptive level, the term avoids interfering with established, treatment-oriented classifications while offering a shared conceptual reference point across orthopedic subspecialties.

### 4.6. Limitations

This study has limitations. The analysis was based on published terminology rather than on patient-level data, and therefore cannot assess the appropriateness of terminology in individual clinical contexts. Additionally, the proposed concept of artificial fracture has not yet been validated through consensus methodologies or applied prospectively in clinical registries.

In addition, the present analysis focused on terminology usage as reported in published literature and did not assess patient-level data or clinical decision-making processes. As a consequence, the study cannot evaluate whether specific terminological choices directly influence treatment strategies or clinical outcomes. Furthermore, the review was limited to articles published in English-language indexed databases, which may have excluded region-specific terminology used in local journals or registries. Finally, the proposed transversal descriptor has not yet been formally tested in prospective registries or validated through consensus methodologies. These limitations are inherent to the conceptual and exploratory nature of the study and further highlight the need for future validation through structured consensus processes and real-world data applications.

## 5. Conclusions

Terminology describing fractures associated with orthopedic implants remains fragmented and inconsistent across the literature. The term **“periprosthetic fracture”** continues to dominate in studies focusing on hip and knee arthroplasty, reflecting the influence of established frameworks such as the Vancouver and Unified Classification Systems [16,17,18,19]. However, this term’s anatomical specificity limits its application to non-arthroplasty scenarios. In contrast, descriptors such as **“peri-implant,” “implant-related,”** and **“hardware-related”** fractures are more frequently used in the context of trauma fixation and spinal or upper-limb surgery, where the implants involved are not prosthetic in nature.

While internally coherent within specific clinical domains, this terminological heterogeneity poses challenges when implant-influenced fractures are analyzed across anatomical sites, registries, and research settings. The lack of a universal lexicon leads to misclassification in registries, inconsistencies in coding, and barriers to large-scale meta-analyses. Moreover, the semantic disunity complicates the integration of orthopedic data into digital health systems, where artificial intelligence and natural language processing increasingly depend on standardized terminology.

The present review identified a persistent gap between **site-specific classification systems**, which provide detailed local frameworks, and the **need for a transversal descriptor** applicable across all implant types and anatomical regions. In response to this gap, we propose the concept of an **“artificial fracture”**—a unifying, higher-order term encompassing any fracture whose morphology, biomechanics, or management are significantly influenced by the presence of an implant.

The “artificial fracture” descriptor is not designed to replace existing systems, but rather to **complement them** as a semantic bridge. It operates at a higher conceptual level, linking diverse anatomical sites and implant types under a common umbrella, while preserving the precision of local classifications such as Vancouver or AO/OTA UCS. By introducing this transversal meta-category, it becomes possible to establish a shared vocabulary across orthopedic subspecialties, improving both the clarity of clinical discourse and the comparability of research findings.

The proposed framework offers several potential advantages:

- **Enhanced terminological clarity:** Provides a coherent language for describing implant-influenced fractures regardless of anatomical site.
- **Improved research comparability:** Facilitates multicenter studies and meta-analyses by standardizing descriptors across domains.
- **Increased coding reliability:** Simplifies classification in hospital information systems and national registries, reducing variability in documentation.
- **Support for multidisciplinary collaboration:** Creates a shared lexicon among orthopedic surgeons, trauma specialists, radiologists, rehabilitation physicians, and researchers.
- **Compatibility with digital systems:** Enables better data mapping for machine learning, natural language processing, and registry-based epidemiology.

Beyond its academic and clinical implications, the “artificial fracture” concept also has relevance for **medical education and policy**. A unified descriptor can streamline teaching by providing students and residents with an overarching conceptual framework that integrates both arthroplasty- and trauma-related fractures. From a public health standpoint, harmonized terminology facilitates accurate surveillance of implant-associated complications and supports the development of international quality indicators for orthopedic outcomes.

### 5.1. Future Directions

The successful implementation of this terminology requires **formal validation** through structured international collaboration. Future efforts should focus on:

1. **Delphi consensus studies** involving key opinion leaders and orthopedic societies (e.g., AO/OTA, ISFR, EFORT, ICS) to assess clarity, acceptability, and applicability.
2. **Registry-based pilot testing** to evaluate coding feasibility and real-world interoperability in electronic data systems.
3. **Cross-linguistic harmonization** to ensure adaptability in non-English-speaking settings, preserving the universality of the term.
4. **Integration into digital health standards** such as ICD-11, SNOMED-CT, and national arthroplasty registries, enabling future data-driven analytics.
5. **Educational dissemination** through textbooks, conferences, and online modules to promote consistent usage among clinicians and trainees.

#### Relevance to Muscle and Musculoskeletal Research

Beyond its orthopedic and terminological implications, this work is directly relevant to muscle and musculoskeletal research. Fractures occurring in the presence of orthopedic implants substantially alter load transmission, muscle force distribution, and neuromuscular coordination, thereby influencing functional recovery and tissue adaptation. A unified and clearly defined terminology for implant-influenced fractures facilitates consistent communication and data integration in studies investigating muscle–bone–implant interactions, rehabilitation outcomes, and biomechanical modeling. By supporting cross-anatomical comparability and registry-level harmonization, this conceptual framework may enhance the interpretability of muscle-related research conducted in mechanically and biologically altered skeletal environments, in line with the interdisciplinary and translational focus of Muscles.

From a musculoskeletal perspective, implant-influenced fractures represent a unique biomechanical condition in which altered load transfer, modified lever arms, and changes in muscle activation patterns may affect functional recovery. Clear and consistent terminology facilitates the interpretation and comparison of studies investigating muscle strength, coordination, and rehabilitation outcomes after fracture in the presence of implants. By supporting semantic harmonization across anatomical regions and implant types, a transversal descriptor may improve data integration in musculoskeletal research, particularly in studies combining orthopedic, biomechanical, and rehabilitation-focused outcomes.

### 5.2. Final Remarks

By addressing the current fragmentation of nomenclature, the concept of an **“artificial fracture”** represents a practical, inclusive, and forward-looking step toward linguistic and conceptual unification in orthopedic traumatology.

It reflects the evolution of orthopedic practice from a site-specific to a system-based understanding of fracture mechanisms, where the presence of an implant fundamentally alters both pathophysiology and management.

Adopting this term may ultimately enhance **scientific communication**, **research comparability**, and **patient care quality**—aligning orthopedic terminology with the needs of a modern, data-driven healthcare environment.

## Data Availability

No new data were created or analyzed in this study.

## Supplementary Materials

The following supporting information can be downloaded at: https://www.mdpi.com/article/10.3390/muscles5010007/s1, Table S1: PRISMA 2020 checklist. Reference [20] is cited in the supplementary materials.
